# Abnormal Uterine Bleed in a Postmenopausal Woman With the Use of Escitalopram

**DOI:** 10.7759/cureus.23432

**Published:** 2022-03-23

**Authors:** Akshita Yadav, Brandon S Bharat, Stephanie Montrose

**Affiliations:** 1 Family Medicine, Ross University School of Medicine, Bridgetown, BRB; 2 Family Medicine, Clarkston Medical Group, Clarkston, USA

**Keywords:** escitalopram, case report, abnormal uterine bleeding, antidepressant, selective serotonin reuptake inhibitor, lexapro, postmenopausal woman

## Abstract

Selective serotonin reuptake inhibitors (SSRIs) are among the most widely used antidepressants worldwide. They are an effective first-line treatment for depression. Common side effects can be quickly remediated by switching to a different drug, making it easy to miss rare side effects and even causing them to go underreported. This case study examines an instance of uterine bleeding in a postmenopausal woman after starting an antidepressant. A detailed history determined that the medication was the only noticeable change in her daily routine before the onset of bleeding, making it the likely cause. Due to the high index of suspicion, the medication was discontinued, and it was apparent that the bleeding ceased. This phenomenon demonstrated the role of serotonin in potentiating the coagulation cascade. Research on this topic is limited, but there have been other reported cases of similar findings in the past.

## Introduction

Depression is a common and complex psychiatric condition that creates challenges for individuals and the physicians trying to treat them. In recent decades, the global prevalence of depression has been steadily increasing. Approximately 20% to 25% of women and 7% to 12% of men will experience clinical depression or depressive symptoms in their lifetime [1]. This can be due to the death of a loved one, loss of a job, the ending of a relationship, increased amounts of stress, hospitalization, a co-existing illness, etc. [2]. Individuals can experience a multitude of symptoms, including, but not limited to, depressed mood, anhedonia, fatigue, guilt, low energy, decreased concentration, changes in sleep, loss of appetite, weight loss or gain, suicidal thoughts, and more. Each person is different and can exhibit a unique combination of symptoms, which can make diagnosing a patient challenging.

## Case presentation

A 54-year-old postmenopausal Caucasian female, with a history of systemic lupus erythematosus and chronic joint pain, presented via telehealth for grief counseling. Five months ago, following the passing of her daughter, she continuously experienced depressive symptoms, including a labile mood, poor affect, trouble sleeping, anhedonia, racing thoughts, and decreased appetite. On presentation, she seemed sad and was tearful but without physical discomfort. At this initial visit, the importance of a psychologist was discussed, and a referral was provided. She was prescribed alprazolam 0.25 mg and citalopram 20 mg and instructed to follow up in one month.

At her next appointment, she described visiting the psychologist weekly and was finding it helpful. Her appetite had improved, but she endorsed no improvement in her other symptoms since her last visit. At this time, citalopram 20 mg was discontinued, and she was started on escitalopram 20 mg with instructions to follow up in a month.

At her monthly follow-up, she described her sleep had improved, and she rarely required her alprazolam 0.25 mg. At this visit, she was more talkative, but she seemed to be in physical discomfort. Upon questioning, she divulged that for the past week, she experienced bleeding similar to her previous menstrual cycles. She has not had a regular period for one year and was currently postmenopausal. Before menopause, she had no history of abnormal menstrual cycles, intermenstrual bleeding, or menometrorrhagia. At this visit, her symptoms included heavy bleeding with clots, requiring the daily use of pads and frequent changes. Aside from the bleeding, there were no other complaints at this visit. On physical examination, she had conjunctival pallor and was tachycardic. All other vital signs were within normal limits. She denied any trauma, recent sexual activity, signs of infection, alcohol consumption, smoking, recreational drug use, and exercise. The only new addition, within the past month, was her recently prescribed escitalopram 20 mg. A brief review unveiled that in rare cases, selective serotonin reuptake inhibitors (SSRIs), such as escitalopram, have been linked to abnormal uterine bleeding that stopped after the cessation of the offending agent [3]. Therefore, escitalopram 20 mg was discontinued, and venlafaxine 75 mg was prescribed for her depression. The patient agreed to monitor her bleeding with the expectation that it should subside within one week and instructions to make an urgent appointment if it persisted or worsened. No further visits were necessary since her bleeding resolved within a few days.

## Discussion

The prevalence of depression increases each year along with antidepressant use. Between 2015 and 2018, 13.2% of individuals over 18 years old reported taking antidepressants within the past 30 days. Medication use was higher among women (17.7%) in comparison to men (8.4%) [4]. Before understanding how antidepressants such as SSRIs work, it is crucial to understand what causes depression. The pathophysiology of depression remains unclear, but the most prevalent theory revolves around neurotransmitters. The “Monoamine Hypothesis” postulates that the concentration of monoamines, such as serotonin, is decreased at synapses within the brain in those with depression [5]. This neurotransmitter is considered to play a role in controlling positive affect and is sometimes referred to as a “happy hormone” [6]. This theory was further supported when the use of SSRIs improved depressive symptoms. SSRIs increase the level of serotonin within synapses by inhibiting the reuptake of the neurotransmitter at the synapse. The medication does this by inhibiting the serotonin transporter in the presynaptic axon terminal, leading to a greater concentration of serotonin in the synaptic cleft and stimulating the postsynaptic receptors [7]. As with any medication, SSRIs can cause adverse effects in a patient. Side effects include increased heart rate, dry mouth, nausea/vomiting, diarrhea, hepatotoxicity, seizure, sexual dysfunction, weight gain, hyponatremia, sweating, and abnormal bleeding [8]. The risk of upper gastrointestinal (GI) bleeding is well-documented but bleeding at other locations has been less commonly described. Unfortunately, the patient described in this case report experienced such bleeding from her uterus.

In a cross-sectional study conducted by Uguz et. al., it was found that the incidence of menstruation disorders in a group of 1432 women taking antidepressants was 14.5% [9]. It is hypothesized that symptoms of bleeding are related to the minor, yet essential, role that serotonin has during hemostasis. The process of platelet aggregation is induced by adenosine diphosphate, and serotonin dose-dependently aids in enhancing the effect [10]. Platelets are unable to synthesize serotonin and acquire it from the bloodstream. Just as SSRIs prevent the reuptake of serotonin at synapses, they also prevent uptake into platelets [11]. If platelets are unable to store serotonin, they will be unable to release it when required during aggregation. Releasing serotonin is vital, as it attaches to P-selectin receptors on platelet membranes, encouraging further aggregation and eventual thrombus formation [10]. Therefore, once the antidepressant is discontinued, platelets will again use serotonin and the bleeding should cease.

## Conclusions

This case report proved to be a unique presentation, as it caused a postmenopausal woman to experience menstrual bleeding again. Bleeding in the upper GI system is commonly documented, but due to the mechanism with which SSRIs interfere with hemostasis, it is plausible to have a chance of bleeding anywhere. An explanation for this could be that people do not think of SSRIs as often when creating differential diagnoses for bleeds. In this case report, the patient was postmenopausal so it was easy to recognize a change in her body. A younger patient with regular menstrual cycles may not be as quick to notice a problem, as any irregularities may be deemed insignificant. Therefore, it is imperative that the relationship between SSRI use and uterine bleeding be further investigated, considering the rates of depression and pharmacotherapy are steadily rising. Patients and physicians should be aware of risks no matter how small so that symptoms do not go unnoticed and are promptly managed.
